# Indocyanine Green-based Glow Nanoparticles Probe for Cancer Imaging

**DOI:** 10.7150/ntno.78405

**Published:** 2023-04-09

**Authors:** Neeraj Chauhan, Marco Cabrera, Pallabita Chowdhury, Prashanth K.B. Nagesh, Anupam Dhasmana, Pranav, Meena Jaggi, Subhash C. Chauhan, Murali M. Yallapu

**Affiliations:** 1 Department of Immunology and Microbiology, School of Medicine, The University of Texas Rio Grande Valley, McAllen, TX 78504, United States; 2 South Texas Center of Excellence in Cancer Research, School of Medicine, The University of Texas Rio Grande Valley, McAllen, TX 78504, United States; 3 Department of Pharmaceutical Sciences, University of Tennessee Health Science Center, Memphis, Tennessee 38163, United States; 4 Laboratory of Signal Transduction, Memorial Sloan Kettering Cancer Center, New York, New York 10065, United States

**Keywords:** indocyanine green, near infra-red fluorescent cancer imaging, deep tissue bioimaging, nanoparticle probes, nanoparticles

## Abstract

Indocyanine green (ICG) is one of the FDA-approved near infra-red fluorescent (NIRF) probes for cancer imaging and image-guided surgery in the clinical setting. However, the limitations of ICG include poor photostability, high concentration toxicity, short circulation time, and poor cancer cell specificity. To overcome these hurdles, we engineered a nanoconstruct composed of poly (vinyl pyrrolidone) (PVP)-indocyanine green that is cloaked self-assembled with tannic acid (termed as indocyanine green-based glow nanoparticles probe, ICG-Glow NPs) for the cancer cell/tissue-specific targeting. The self-assembled ICG-Glow NPs were confirmed by spherical nanoparticles formation (DLS and TEM) and spectral analyses. The NIRF imaging characteristic of ICG-Glow NPs was established by superior fluorescence counts on filter paper and chicken tissue. The ICG-Glow NPs exhibited excellent hemo and cellular compatibility with human red blood cells, kidney normal, pancreatic normal, and other cancer cell lines. An enhanced cancer-specific NIRF binding and imaging capability of ICG-Glow NPs was confirmed using different human cancer cell lines and human tumor tissues. Additionally, tumor-specific binding/accumulation of ICG-Glow NPs was confirmed in MDA-MB-231 xenograft mouse model. Collectively, these findings suggest that ICG-Glow NPs have great potential as a novel and safe NIRF imaging probe for cancer cell/tumor imaging. This can lead to a quicker cancer diagnosis facilitating precise disease detection and management.

## Introduction

Cancer incidences are on the rise around the world, including in the United States. According to the American Cancer Society, about 1,958,310 people will be diagnosed with cancer, and 609,820 people will be dying from it in 2023 1. The combination of early diagnosis and surgical removal of tumors is the best effective therapeutic modality. To date, several non-invasive clinical imaging approaches have been implemented for detecting and managing cancer, namely, X-ray, ultrasound, computed tomography, magnetic resonance imaging, positron emission tomography, and single photon emission computed tomography 2. However, these imaging procedures are not only associated with high cost, but also linked to low specificity, sensitivity, resolution, and slow throughput.

Fluorescence imaging has gained attention in both chemical and biological fundamental research due to its high sensitivity, quick response, and simplicity. Cancer detection utilizing near-infrared (NIR) fluorescent agent(s) allows to assess optical properties of tissues 3. NIR fluorescent imaging offers real-time monitoring of biological processes in living tissues with high spatial-resolution without having to use any ionizing radiation 4. Additionally, biological tissues show reduced autofluorescence and light absorption in the NIR spectral region (750 - 900 nm) 5. Moreover, fluorescence optical imaging performed with NIR agents does not use any radioactive components and/or any ionizing radiation, thus, it can be used repeatedly at a reduced cost, and be applicable in both adults and young patients 6. Therefore, there is a considerable demand to develop NIR fluorescent probes that enable tissue imaging, offering a bench-to-bedside opportunity for cancer diagnosis and imaging.

To date, several NIR fluorophores have been investigated for NIR fluorescence imaging. However, indocyanine green (ICG) and methylene blue (MB), are the approved NIR fluorescence contrast agents/dyes for clinical use by the Food and Drug Administration (FDA) and the European Medicines Agency. Nevertheless, both ICG and MB are non-specific NIR dyes with no tumor specific binding/targeting capacity 7. MB is only safe at lower doses (<2 mg/kg) and can induce severe adverse effects with higher doses, including coronary vasoconstriction, arrhythmias, and hemolytic anemia in patients with renal disease. MB has an excitation peak of approximately 700 nm, therefore, has a less tissue penetration capacity and background tissues show additional autofluorescence 7, 8. Conversely, ICG is a safe dye for human use as the adverse effects rate is very low and mainly associated with hypersensitivity reactions 9. ICG has an excitation range of 740-800 nm and an emission of 800-860 nm, thus can penetrate through deep and dense tissues with minimal background autofluorescence 10. ICG dye accumulates in hyperpermeable tissues prior to surgery which discriminates the tumor from non-tumor tissue 11. Currently, there are about 194 clinical trials based on ICG imaging for cancers 12. ICG has low bioavailability and distribution in the body (about 3-4 minutes) 13 and majority of it being excreted by the liver 14. Some studies have shown identical *in vitro* and *in vivo* toxicity when used at a higher dose than what was used clinically 15. In addition, ICG undergoes photobleaching when exposed to NIR light, concomitantly having other challenges such as stability issues for a prolonged period and precipitation at higher concentrations 16. Therefore, there is an unmet urgent clinical need for the development of cancer cell-specific ICG imaging agent*.*

A nanoparticle-based system is a route to optimally achieve tumor-specific delivery of ICG. Nanoparticles (NPs) offer passive delivery utilizing enhanced permeability and retention (EPR) mechanism (i.e., passive targeting) that renders agents to penetrate through the leaky tumoral neo-vasculature allowing nanoparticles of therapeutic agents/dyes to circulate longer and deliver them to specific tumor sites 17, 18. There have been studies that explore the use of ICG-loaded/encapsulated nanoprobes, allowing efficient and prolonged imaging 19. ICG nanocarriers have the potential to increase the specificity, efficiency, and biosafety of imaging techniques 20. Such nanocarriers demonstrate accumulation at tumor sites 21. For instance, Pegsitacianine (micellar polymer-ICG tracer, pH-responsive) from OncoNano Medicine, Inc. 22, 23, and ICG-Chlorin-e6 nanocluster 24, poly(caprolactum)-ICG micelles 25, and OTL38 (NIRF conjugated dye with a ligand targeting folate receptor α) were developed recently 26, 27. Tumoral delivery by active targeting *via* attaching ligands such as polymers (polyethylene glycol or pluronics), cationic peptides, aptamers, and antibodies on nanoparticles, have been employed 28. Such strategy provides active targeting to the receptors that are discretely unregulated on cancer cells, thus allowing receptor-mediated endocytosis that provides cell-specific adherence and uptake of nanoparticles 29. Therefore, following the novel self-assembly approach, we have engineered a nanoconstruct *via* self-assembly process that is comprised of poly(vinyl pyrrolidone) (PVP), ICG, and tannic acid (TA) (named as indocyanine green-based glow nanoparticles probe, ICG-Glow NPs). We have evaluated this ICG-Glow NPs probe for superior cancer/tumor cell-specific targeted binding and NIRF imaging.

## Material and Methods

### Chemicals, reagents, and cell culture

All solvents, chemicals, and reagents were purchased from Sigma Aldrich Corporation (St. Louis, MO, USA) and used as such, unless otherwise stated. All cancer cell lines were purchased from American Type Culture Collection (ATCC) (Manassas, VA, USA). Normal human epithelial [human embryonic kidney-293 (HEK-293) and human pancreatic epithelial nestin-expressing (HPNE)], breast cancer (MCF-7), liver cancer (HEPG-2), prostate cancer (PC-3), and pancreatic cancer (AsPC-1) cell lines were maintained in DMEM, EMEM, and RPMI media (Gibco, Gibco laboratories, Gaithersburg, MD, USA), containing 10% heat inactivated FBS (Atlantic Biologicals, Lawrenceville, GA, USA) and 5 mL 1X antibiotic/antimycotic (Sigma, St. Louis, MO, USA) supplements. Cell lines were cultured at 37 ºC under a humidified atmosphere of 5% CO_2_.

### Preparation of ICG-Glow NPs

ICG-Glow NPs were prepared using the self-assembly method followed by solvent evaporation 30-34. For this, PVP (100 µL of 1 mg/mL in water) and ICG (NIR dye, 20 µL of 1 mg/mL in water) solutions were added together with 780 µL of miliQ water in an 8 mL glass vial under continuous stirring condition at 700 rpm on a stir plate for 4 h. After 4 h, 100 µL of TA (1 mg/mL in water) solution was added dropwise to cloak PVP-ICG construct and stirred overnight to ensure the self-assembly cloak of TA that led to the formation of ICG-Glow NPs. For comparison, coumarin 6 (C6, fluorescent dye) (1 mg/mL in acetone) or 1,1′-Dioctadecyl-3,3,3′,3′-tetramethylindocarbocyanine perchlorate (DiL, NIR dye) (1 mg/mL in acetone) were also employed instead of ICG. Blank PVT nanoparticles were prepared following the similar protocol without adding the dyes.

### Physico-chemical characterization

**Dynamic Light Scattering:** Particle size distribution and zeta potential (surface charge) of freshly prepared ICG-Glow NPs were measured through a Dynamic Light Scattering (DLS) system (Zetasizer Ultra, Malvern Panalytical, Malvern, United Kingdom). For this, samples (150 μL sample in 3 mL Milli-Q water for size and 50 μL sample in 1 mL 1X PBS for charge) were ultrasonicated for ~ 45 seconds and transferred into a clear plastic cuvette (size distribution) or in a capillary cell (zeta potential) for measurements. All measurements were performed in triplicates at 25 °C.

**Spectral Analysis:** Biochemical characterization of ICG-Glow NPs formulation was assessed by utilizing a UV-Vis spectrophotometer (Varioskan LUX Multimode Microplate Reader, Thermo Fisher Scientific, Waltham, MA, USA). Free ICG and ICG-Glow NPs were prepared at 100 μg concentration and assay was carried out at room temperature in 96‐well black microplates. The absorption spectral measurements were recorded from 300-900 nm. Recorded data was analyzed using Skanlt Software RE 6.02.

**Transmission electron microscopy:** The visualization of ICG-Glow NPs was achieved by utilizing JEOL 2000EX transmission electron microscope (TEM) (JEOL Ltd., Tokyo, Japan). The ICG-Glow NPs solution was initially sonicated and smaller volume was deposited on the formvar carbon-coated side of a 150-mesh standard TEM grid (Electron Microscopy Sciences, PA, USA). Upon removing excessive solution from grid, nanoparticles were negatively stained with 1% (wt) uranyl acetate (Electron Microscopy Sciences) solution. The air-dried nanoparticles were visualized under TEM using an AMT camera system at a direct magnification of 100,000×.

### *In vitro* ICG release

*In vitro* release profile of ICG from ICG-Glow NPs was analyzed using dialysis membrane bag-based drug release method. For this, 1 mL of 100 µg ICG containing ICG-Glow NPs suspension was placed in a dialysis membrane bag (SnakeSkin Dialysis Tubing, molecular weight cut off size, 3.5 kDa, Thermo Scientific, Waltham, MA, USA) which was submerged in 10 mL PBS-T (1X PBS containing 0.1% (wt/vol) Tween-80) in a 50 mL conical falcon tube placed on an orbital shaker at 37°C with continuous rotating at 150 rpm. Outside samples from falcon tube were collected at different time points [0.4 day (3 h), day 1 (24 h), day 3 (72 h), day 5 (120 h), and day 7 (168 h)] and released ICG from ICG-Glow NPs was measured by a UV-Vis spectrophotometer (Varioskan LUX Multimode Microplate Reader). A linear calibration curve in the range of 0.5 to 100 µg/mL was obtained as identical to dye release process and utilized for calculating the released ICG from ICG-Glow NPs.

### Fluorescence Imaging on filter paper and chicken breast

We have utilized 1 mg/mL ICG and ICG equivalent ICG-Glow NPs for this experiment. A 5 µL drop was placed onto a Whatman filter paper containing different ICG concentrations (7.5, 10, 12.5, 15, 17.5, and 20 µg). The fluorescent optical images were acquired with IVIS spectrum imaging system (PerkinElmer, Waltham, MA) using filters from 780 nm to 845 nm for ICG. Regions of interest (ROIs) of the corresponding dots/areas were drawn over the optical images, and the average radiant efficiencies from 3 individual experiments were calculated, which provided the quantification of the fluorescence intensities. For comparison purpose, we have also utilized other dyes such as C6 and DiL. The acquisitions were made using filters from 460 nm to 520 nm for C6, and from 520 nm to 570 nm for DiL.

For *ex vivo* fluorescence imaging evaluation, ICG and ICG containing ICG-Glow NPs were prepared as mentioned above and similarly a 5 µL drop was placed onto a freshly purchased chicken breast containing different ICG concentrations (7.5, 10, 12.5, and 15 µg). These chicken breasts with ICG-Glow NPs were imaged using IVIS imaging system as mentioned earlier for the ICG fluorescence.

### Hemo-and cellular-biocompatibility

We performed a hemolysis assay to evaluate the hemocompatibility profile (hemocompatibility with blood) of ICG-Glow NPs following our previously optimized and published protocol 35. Briefly, human whole blood from a single donor was procured from Innovative Research, Inc. (IWB1K2E10ML, Novi, MI, USA). 10 mL blood was centrifuged at 2000 rpm for 10 minutes to collect red blood cells (RBCs) as pellet and resuspended in 10 mL phenol red free RPMI-1640 cell culture medium. RBCs (100 μL) were then treated with free ICG and ICG-Glow NPs (10, 25, 50, and 100 µg), sodium dodecyl sulfate (SDS, positive control, 100 µg), and 1XPBS (negative control, 100 µg) in Eppendorf tubes for 2 h at 37 °C. Next, treated RBCs were centrifuged for 5 minutes at 1000 rpm and supernatant was used for optical density measurement at λ_max_ 570 nm utilizing a multimode microplate reader (Varioskan LUX) while the collected RBCs pellet was saved for qualitative microscopic analysis using a brightfield microscope (EVOS M7000, Invitrogen, Thermo Fisher Scientific, MA, USA) to observe any morphological changes caused by hemolysis. Onto a glass slide, a drop of 5-10 µL RBCs pellet was placed and smeared with another spare slide, secured with a cover slip, and imaged 36. Images were taken at 40X. For investigating possible ultrastructural change in the morphology of RBCs, a scanning electron microscope (SEM) (JCM-7000 NeoScope™ Benchtop, JOEL, Houston) was employed. For this study, RBC-treated cells were fixed in 4% formaldehyde and dehydrated serially with 30%, 50%, 70%, 90%, and 100% ethanol. These cells were deposited on glass slide and were kept for air dry. Afterwards the air-dried samples were gold coated for 2 minutes using DII-29010SCTR smart sputtering coater. The coated samples were then visualized under JCM-7000 NeoScope™ Benchtop SEM by applying 5 kV voltage.

Cellular biocompatibility of ICG-Glow NPs was determined by using an MTS assay [CellTiter96 Aqueous One Solution (Promega, Madison, WI, USA)] with various cancer cell lines. For this assay, HEK-293 [human kidney normal), HPNE (human pancreatic normal), MCF-7 (breast cancer), HEPG-2 (liver cancer), AsPC-1 (pancreatic cancer), and PC-3 (prostate cancer) cells (5000/well density) were plated in 96-well plates and allowed to adhere overnight. Next day, HEK-293 and HPNE cell lines were treated with 10, 25, 50, and 100 µg of ICG and ICG-Glow NPs for 24 h, however, cancer cell lines were only treated with 100 µg free ICG and ICG-Glow NPs for the same period. After indicated time, cells were washed, replaced with 100 µL fresh phenol red free media and imaged using a phase contrast microscope (EVOS M7000). Next, 20 µL/well MTS reagent was added for 2-3 h and plates were incubated at 37 °C. After indicated period, plates were read at 490 nm and absorbency data was represented as normalized to non-treated cells. Experiment was performed three individual times.

### *In situ* tumor specific binding evaluation

Tissue microarrays (TMAs) of various tumor grades and normal adjacent tissues for different cancers (Breast #BR087e, Liver #BC03117a, Pancreatic #T142c, Lung #LC242a, and Kidney #T071b) were commercially purchased from US Biomax, Inc. (Derwood, MD, USA). The binding of ICG and ICG-Glow NPs on human tumor tissues was conducted by following immunohistochemistry (IHC) protocol 37, 38. Briefly, slides were deparaffinized at 62°C for 2 h, rehydrated by different concentrations of alcohol content followed by antigen retrieval. Slides were then incubated with freshly prepared 100 µg/mL ICG or ICG equivalent ICG-Glow NPs using a PAP pen for overnight, followed by three consecutive washes of 10 minutes each and imaged using a fluorescent microscope (EVOS M7000) under CY7 channel with 40X objective lens. All images were captured using the same laser power and gain settings to ensure comparability for intensity analysis. Fluorescence intensities were further quantified using Image J software 37.

### Cellular binding/targeting

Cells were seeded in 4-chamber slides with 100,000 cells/chamber and allowed to adhere overnight. Next day, cells were washed twice with 1X cold PBS and fixed with 4% cold paraformaldehyde for 10-15 minutes at room temperature and stored in 500 µL PBS at 4 °C until further used. Slides were then treated with 20 µg free ICG or ICG-Glow NPs for overnight at 4 °C, washed thrice with 1X PBS and cover slipped using the mounting medium. Slides were stored at 4 °C for at least 1 day prior to imaging for complete drying. EVOS M7000 fluorescent microscope was used to image cells under CY7 channel for ICG binding at 20X.

### *In vivo* tumor targeting

Female athymic nude mice (Jackson Laboratory, Bar harbor, ME, USA) were used to determine tumor targeting efficacy of NPs. This study was conducted according to procedure approved by the University of Tennessee Health Science Center (UTHSC) Institutional Animal Care and Use Committee (IACUC) protocol (ID 18-046.0.). Animals were housed in the animal house at the Cancer Research Building of University of Tennessee Health Science Center (Memphis, TN, USA) and animals had access to water and food ad libitum. The tumor xenografts on hind flanks of mice were established using breast cancer cell line (MDA-MB-231, 2×10^6^ cells) according to our established protocol 32, 39. Three mice per group (ICG or ICG-Glow NPs) was randomly distributed and were treated with either 100 µg ICG solution or 100 µg equivalent ICG containing ICG-Glow NPs by intraperitoneal administration. Under anesthetic condition, mice were imaged at 3, 6, and 24 h by IVIS XRMS Imaging System (Caliper Life Sciences, Waltham, MA) 39, 40. After tumor targeting imaging, mice were sacrificed, and tumors were dissected and *ex vivo* quantification of fluorescence was taken by IVIS XRMS Imaging System.

### Docking Studies

All 16 crystal structure of proteins [ERBB3/HER3 (PDB: 3LMG), CXCR4 (PDB: 4RWS), VEGFR2 (PDB: 2P2H), GSK3B (PDB: 1PYX), P-glycoprotein (PDB: 6C0V), STAT1 (PDB: 1YVL), Estrogen receptor-alpha (PDB: 1A52), ERBB2/HER2 (PDB: 3RCD), CTNNB1 (PDB: 1JPW), Estrogen Receptor Beta (PDB: 5TOA), VEGFR1 (PDB: 3HNG), Androgen Receptor (PDB: 2PIW), VEGFR-3 (PDB: 4BSJ), PSMA (PDB: 7BFZ), JAK2 (PDB: 6E2Q), and MUC1 (PDB: 6BSC)] were retrieved from the RCSB protein data bank. The retrieved structures were clean, prepared and minimized by using BIOVIA Discover Studio. 3D structure of tannic acid (PubChem CID: 16129778) was constructed by using Biovia Discovery Studio. The geometric based algorithm software FireDock docking server was applied for the interaction analysis between all 17-crystal structure of proteins and tannic acid 41.

### Statistical analysis

All statistical calculations were performed using Microsoft excel and GraphPad Prism 9.3.1 (GraphPad Software, Inc., La Jolla, CA, USA). The data are presented as mean ± standard error of mean (SEM) and considered significant if ^*^p-values <0.05.

## Results

**Figure 1:**
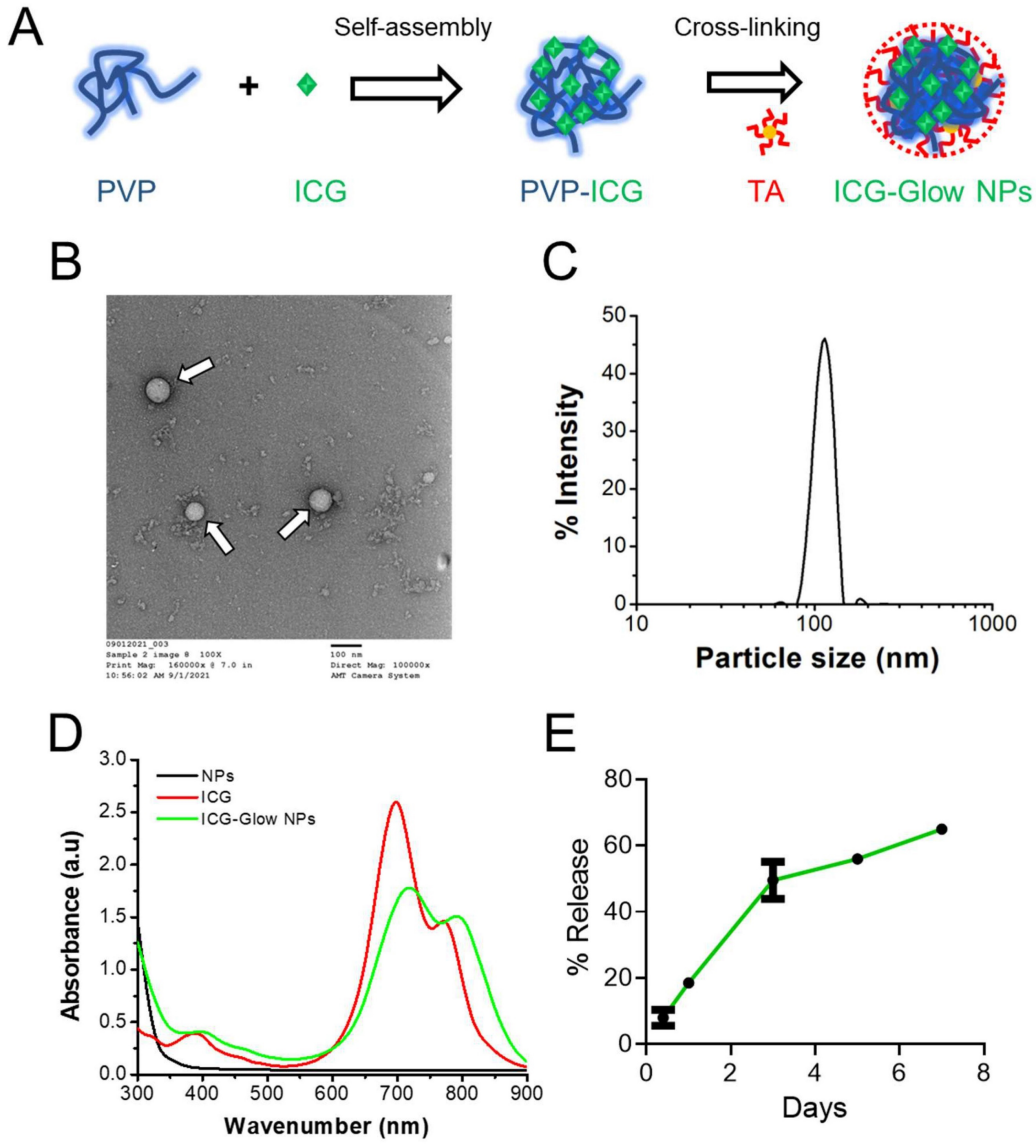
** Generation and physico-chemical characterization of ICG-Glow NPs. A)** Schematic representation of preparation route of ICG-Glow NPs. **B)** Transmission electron microscopic image of ICG-Glow NPs. Bar indicates 100 nm. Image was acquired at 100X print magnification. **C)** Particle size and distribution of ICG-Glow NPs measured by DLS. **D)** UV-Vis spectra of free ICG, ICG-Glow NPs, and blank PVT NPs. **E)** ICG release from ICG-Glow NPs.

Considering the importance of the development of a NIR fluorescent probe, we propose a facile polymer self-assembly approach 42 (**Figure 1A**) which may offer a solution for such interface imaging characteristic issues. We chose a novel self-assembly composed of PVP and ICG which is cross-linked and stabilized/cloaked with TA layers (ICG-Glow NPs). The selection of PVP, ICG, and TA is because: 1) PVP is a synthetic water-soluble polymer, widely employed as a binder in many pharmaceutical tablets and eye drops and 2) ICG is an FDA-approved NIR fluorescent dye, and 3) tannic acid, a natural polyphenol molecule, has shown drug carrier properties 43-45 and has been tested for controlled drug release and improved therapeutic benefits in 46-49 *in vitro* and *in vivo* mouse 45 models, and for its inherent cancer cell targeting characteristics. The objective of this study is to evaluate NIR fluorescent characteristics and tumor-specific targeting and imaging capabilities of ICG-Glow NPs.

### Characterization of ICG-Glow NPs

The physico-chemical characterization of self-assembled ICG-Glow NPs was performed by determining their particle size, morphology, surface charge, and composition utilizing TEM, DLS, and spectral instruments. TEM images of ICG-Glow NPs showed a spherical shape nanoparticle (**Figure 1B, pointed with white arrows**). DLS data indicated that average particle size and surface charge (zeta potential) of ICG-Glow NPs were 125±1.1 nm (**Figure 1C**) and -35±2.6 mV, respectively.

Further, UV-Vis spectral analysis of ICG-Glow NPs revealed that absorbance peaks at 400 nm, 720 nm, and 790 nm appeared due to the presence of ICG in the formulation **(Figure 1D, green line).** Without ICG, PVP-TA NPs did not show any absorbance peaks **(Figure 1D, black line).** Furthermore, a sustained release of ICG was noticed from ICG-Glow NPs (**Figure 1E**). About 18±0.5% and 65±0.3% ICG release was observed in day 1 and 7, respectively.

### ICG-Glow NPs exhibit NIR fluorescence characteristic

**Figure 2:**
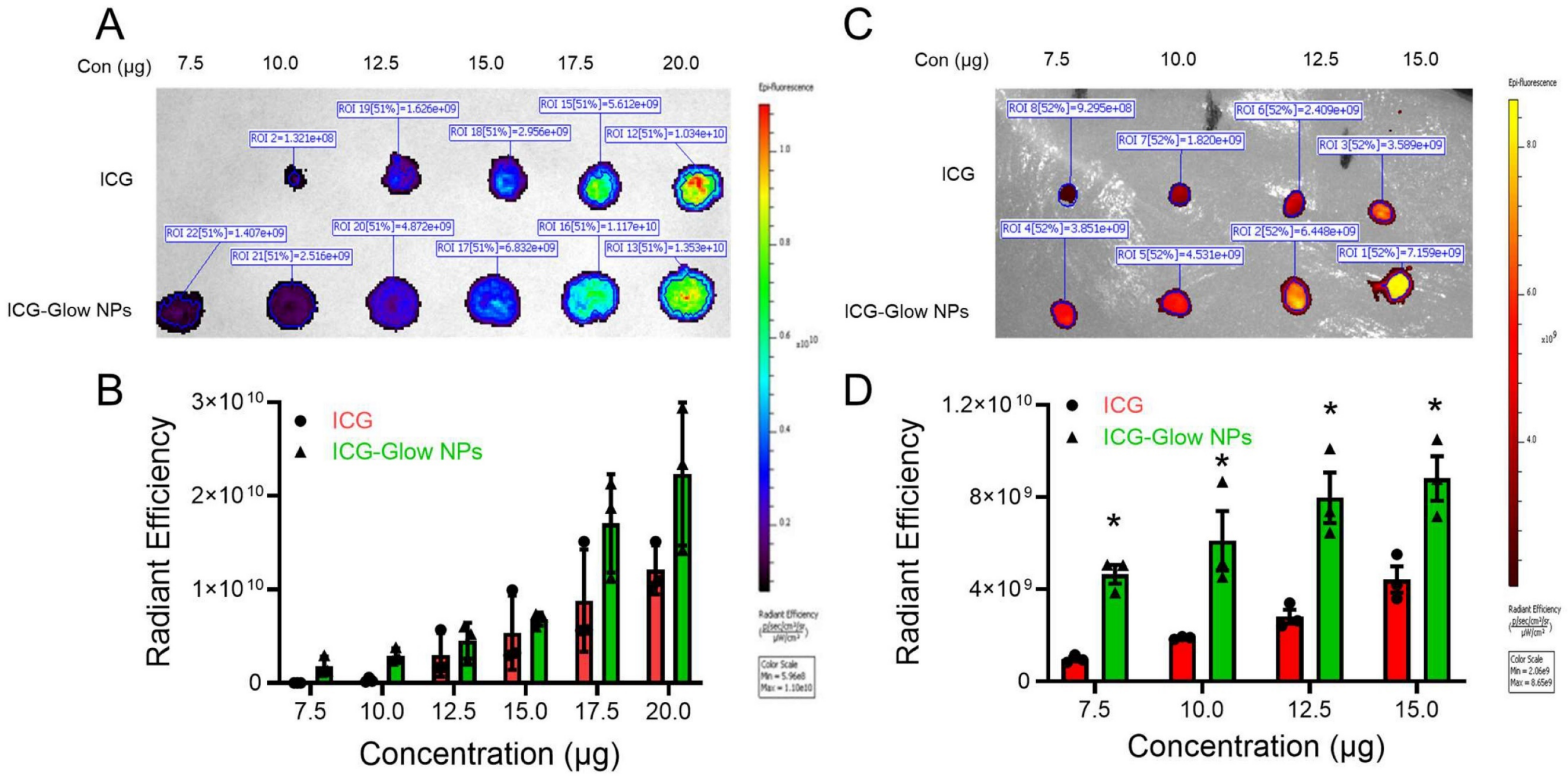
** NIR fluorescence imaging characteristics of ICG-Glow NPs. A-B)** ICG-Glow NPs fluorescence measurement shown on filter paper image, and quantitative analysis of the fluorescence signals ROI (radiation efficiency = photons/sec/cm2/sr) of samples. Data represents the average of three individual experiments. Error bars show SEM, n=3. **C-D)** NIR fluorescence of ICG-Glow NPs on chicken breast tissue and fluorescence ROI quantification. Data represents the average of three individual experiments. Error bars show SEM, n=3. *p<0.05.

To evaluate the NIR fluorescence efficiency of ICG-Glow NPs, we utilized an IVIS spectrum imaging system. For this, a 5 µL drop of different concentrations (7.5 - 20 µg) was placed on a filter paper and imaged at NIR region. It is evident that there is an improved fluorescence intensity in a concentration-dependent manner for ICG-Glow NPs compared to free ICG dye (**Figure 2A-B**). It is worth noting that difference between different concentrations was more prominent for ICG-Glow NPs as 2.9 × 10^9^ > 3.9 × 10^9^ > 5.8 × 10^9^ > 6.6 × 10^9^ > 1.5 × 10^10^ > 2.0 × 10^10^ RFU for 7.5, 10, 12.5, 15, 17.5, and 20 µg, respectively. It is also important to note that like ICG-Glow NPs, C6-Glow NPs (PVP-C6-TA NPs) and DiL-Glow NPs (PVP-DiL-TA NPs) also exhibited higher fluorescence radiant efficiency at all concentrations tested compared to their free dye solutions **(Supplementary Information, Figure S1).** This result suggests that ICG-Glow NPs had higher fluorescence than free ICG, which indicates that encapsulation of ICG in the ICG-Glow NPs system makes ICG more photostable.

To investigate the NIR fluorescence imaging capacity of ICG-Glow NPs, we evaluated tissue bioimaging utilizing freshly procured chicken breast. Different concentrations (7.5, 10, 12.5, and 15 µg) of free ICG and ICG-Glow NPs were placed (5 µL drop) on top of chicken breasts. Dye was allowed to penetrate deep in thick chicken breast tissues and tissues were imaged using an animal imager. The *ex vivo* fluorescence was recorded as radiant efficiency. It was apparent from the results that ICG-Glow NPs showed a concentration dependent higher fluorescence when compared to free ICG (**Figure 2C-D**). Interestingly, fluorescence intensity recorded for ICG-Glow NPs (3.9 × 10^9^ RFU) at the lowest concentration of 7.5 µg was almost similar to free ICG (3.6 × 10^9^ RFU) at 15 µg which was the highest concentration which denotes that ICG-Glow NPs enable ICG to be a suitable and promising tool for deep tissues NIR bioimaging even at low dose.

### ICG-Glow NPs show hemo- and cellular biocompatibility

**Figure 3:**
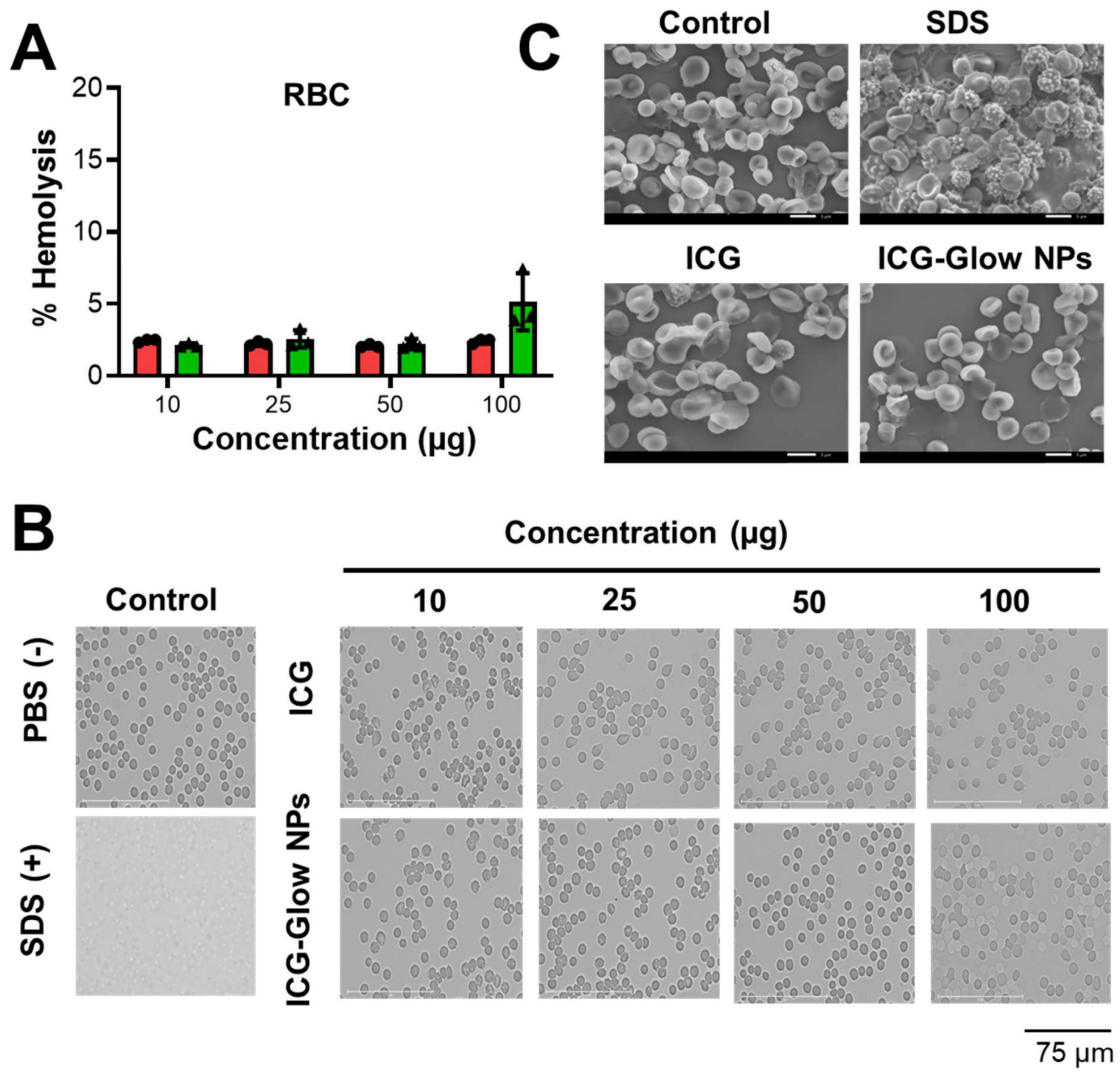
** Hemocompatibility of ICG-Glow NPs. A-B)** Hemocompatibility with Human Red Blood Cells. **A)** Hemolysis of RBCs upon incubation with 10-100 µg/mL ICG or ICG-Glow NPs and SDS serves as positive control in this study. Error bars show SEM, n=3. **B)** Representative microscopic images of RBCs treated with different concentrations of ICG and ICG-Glow NPs. 1X PBS and SDS were used as negative and positive control groups at 100 µg concentration. Images are representative of triplicates. Images were taken using 40X objective lens. **C)** Representative SEM images of RBCs treated with negative control (1X PBS), positive (SDS), ICG, and ICG-Glow NPs. Concentration is equivalent to 100 µg.

It is imperative to determine the compatibility behavior of prepared ICG-Glow NPs with blood prior to its pre-clinical and clinical applications. For this, we executed a red blood cell (RBC) toxicity assay with ICG-Glow NPs following a range of different concentrations. Compared to SDS (positive control), free ICG and ICG-Glow NPs treated RBCs presented a hemocompatible response with minimal to no toxicity effects (**Figure 3A-B**). Negligible hemolysis was noticed for all tested concentration with RBCs (less than ~ 5%), which indicate that the formulations are hemocompatible and consistent with our previous reports 36, 50. For a better assessment of hemocompatibility, SEM images of untreated RBCs and with ICG-Glow NPs treated with RBCs were recorded **(Figure 3C).** SEM images of sodium dodecyl sulfate-treated RBCs (positive control) exhibited rupture of the RBC membrane and/or de-morphed disk shape of cells. While there was no sign of toxicity was evident from healthy smooth oval biconcave disc shape morphology in ICG, ICG-Glow NPs treated RBCs (like in a control RBCs) **(Figure 3C).**

**Figure 4:**
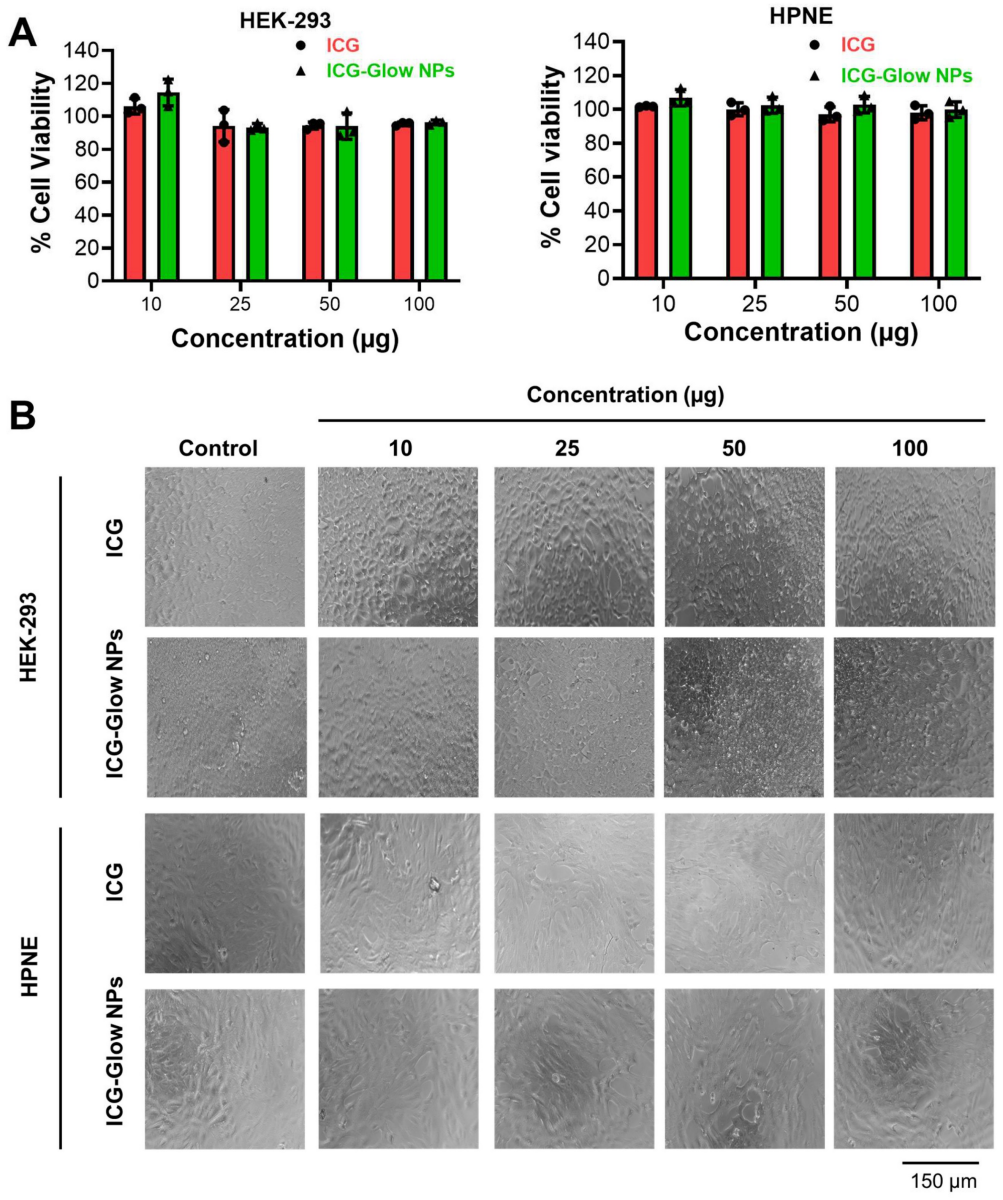
Cellular biocompatibility of ICG-Glow NPs. **A)** Evaluation of cellular compatibility study by MTS cell proliferation assay on human kidney normal (HEK) and human pancreatic normal (HPNE) cell lines. Cell lines were treated with 0-100 µg ICG and ICG-Glow NPs for 24 h and absorbance was measured after MTS reagent supplementation at 490 nm to determine cell viability. Results were normalized to non-treated control cells. Error bars show SEM, n=3. *p<0.05. B) Representative cellular images of treatment groups.

Similarly, the cell viability effect of ICG and ICG-Glow NPs was evaluated by employing an MTS assay using HPNE and HEK-293 human normal cells. It was apparent that minimal to no toxicity was observed up to 100 μg concentration (**Figure 4A-B**).

The cell compatibility study was also tested on MCF-7, HEPG-2, AsPC-1, and PC-3 cancer cells which shows no effect on cell viability (Supplementary Information Figure S2). Together, these results establish that ICG-Glow NPs exhibit superior hemo- and cellular-biocompatibility.

### ICG-Glow NPs introduce tumor/cancer cell specific binding and imaging properties

The affinity of ICG-Glow NPs toward cancer cells/tissues for *in situ* and *in vitro* NIR fluorescence bioimaging was estimated through a binding study. For *in vitro* imaging study, we selected a lower dose (20 µg) as cells were grown in a 2D monolayer. However, a higher concentration of 100 µg was used for *in situ* imaging of TMAs because of thick tumor tissue density.

**Figure 5:**
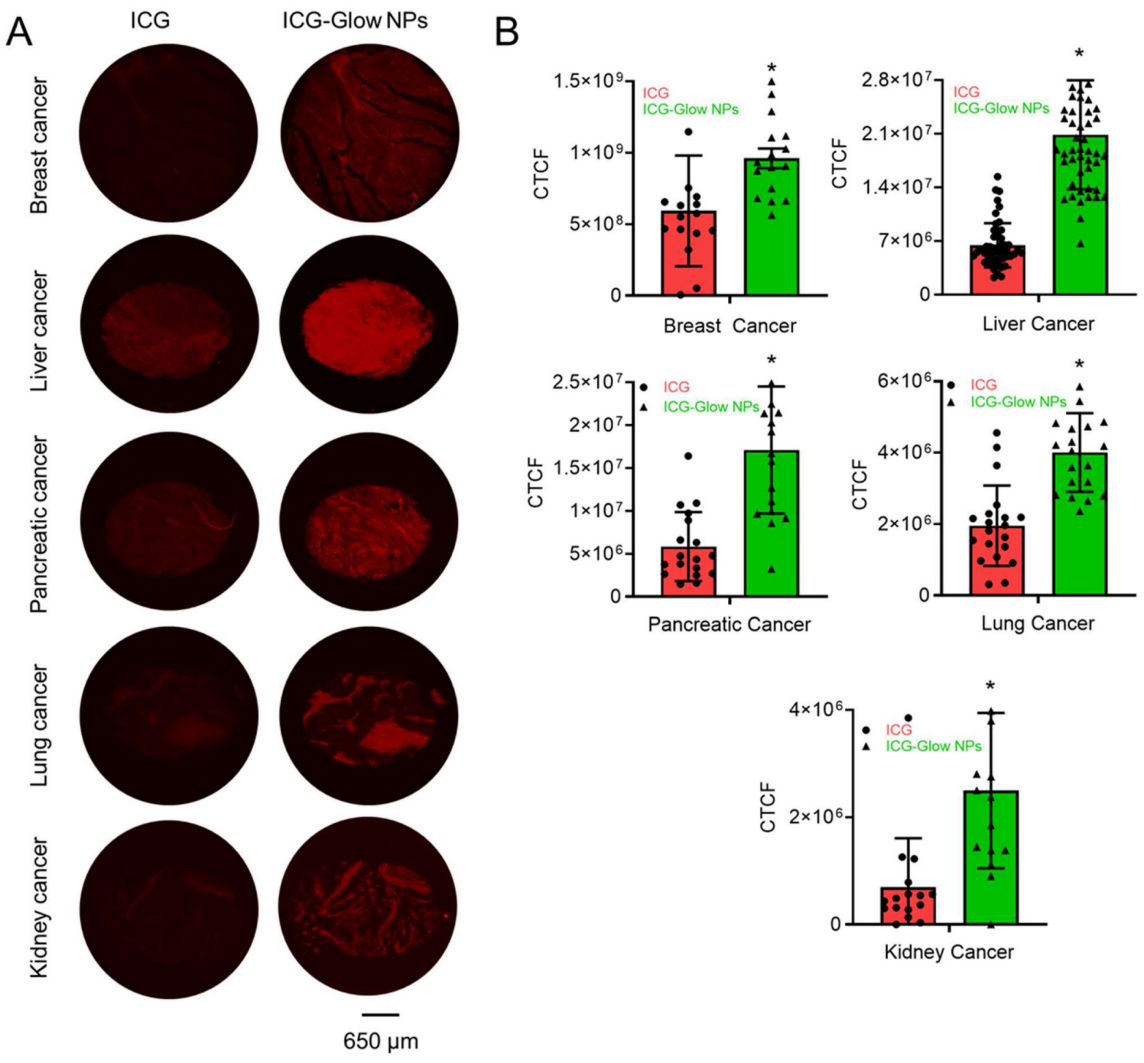
** Clinical significance of ICG-Glow NPs in human cancer. A)** Binding affinity of ICG-Glow NPs was estimated by immunohistochemistry on human cancer tissue microarrays. Breast cancer (n=16), Liver cancer (n=51), Pancreatic cancer (n=18), Lung cancer (n=20), and Kidney cancer (n=16) TMAs were incubated with 100 µg free ICG and equivalent ICG-Glow NPs overnight, following the standard immunohistochemistry procedure. ICG-Glow NPs exhibited significantly higher fluorescence/binding with cancerous tissues than free ICG. Images were taken at 40×. **B)** Quantitative analysis of the fluorescence signals of tissues. Error bars show SEM, *p<0.05.

The *ex vivo* binding and imaging efficacy of ICG-Glow NPs were achieved by using human cancer TMAs. The ICG-Glow NPs in solution were deposited on TMAs overnight for targeted binding. The bound ICG or ICG-Glow NPs on TMAs exhibited fluorescence. As presented in **Figure 5A**, the tumor cores were glowing significantly more with ICG-Glow NPs than free ICG. The quantified fluorescence signal intensities in tumor cores demonstrated that ICG-Glow NPs were ~ 1.6 (Breast), ~ 3.3 (Liver), ~ 3.0 (Pancreatic), ~ 2.1 (Lung) and ~ 2.9 (Kidney) folds higher than free ICG, respectively (**Figure 5B**).

**Figure 6:**
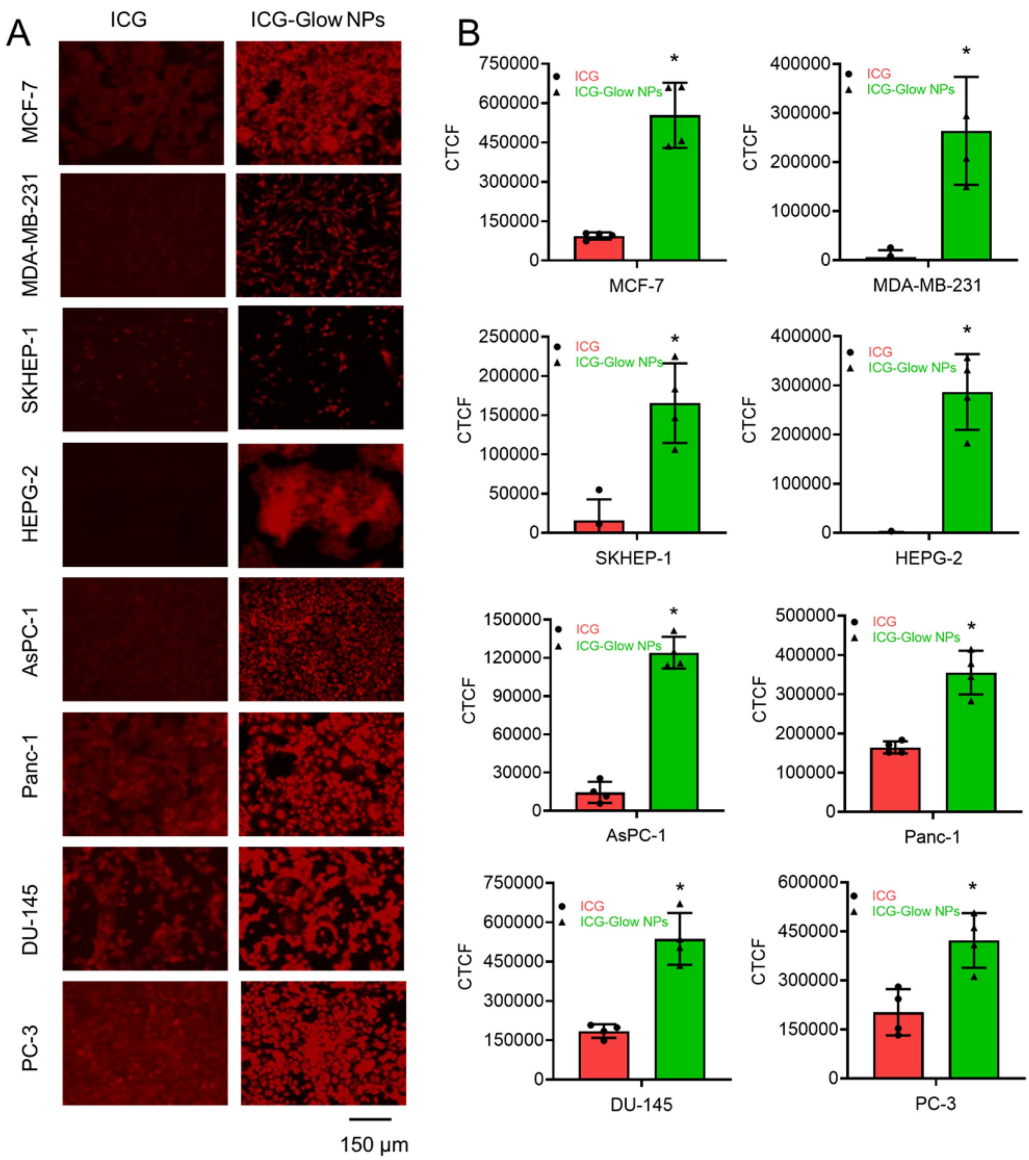
***In vitro* binding affinity of ICG-Glow NPs on various cancer cell lines. A)** MCF-7, MDA-MB-231 (Breast), SKHEP-1, HEPG-2 (Liver), AsPC-1, Panc-1 (Pancreatic), and DU-145, PC-3 (Prostate) cancer cell lines were fixed and then exposed to 20 µg ICG-Glow NPs and free ICG. Representative images were presented. Images were taken at 200×. **B)** Quantitative presentation of fluorescence binding of ICG/ICG-Glow NPs on cancer cells. Error bars show SEM, n=3. *p<0.05.

Furthermore, the cancer cell binding efficacy of ICG-Glow NPs was evaluated on cancer cells. In this assay, NIR fluorescent imaging data revealed that ICG-Glow NPs had more preferential binding affinity to cancer cells compared to free ICG. This phenomenon is observed from the fluorescence images of cancer cells (**Figure 6A**) and quantified fluorescence signal intensities data (**Figure 6B**).

### ICG-Glow NPs target and accumulate at tumor site

**Figure 7:**
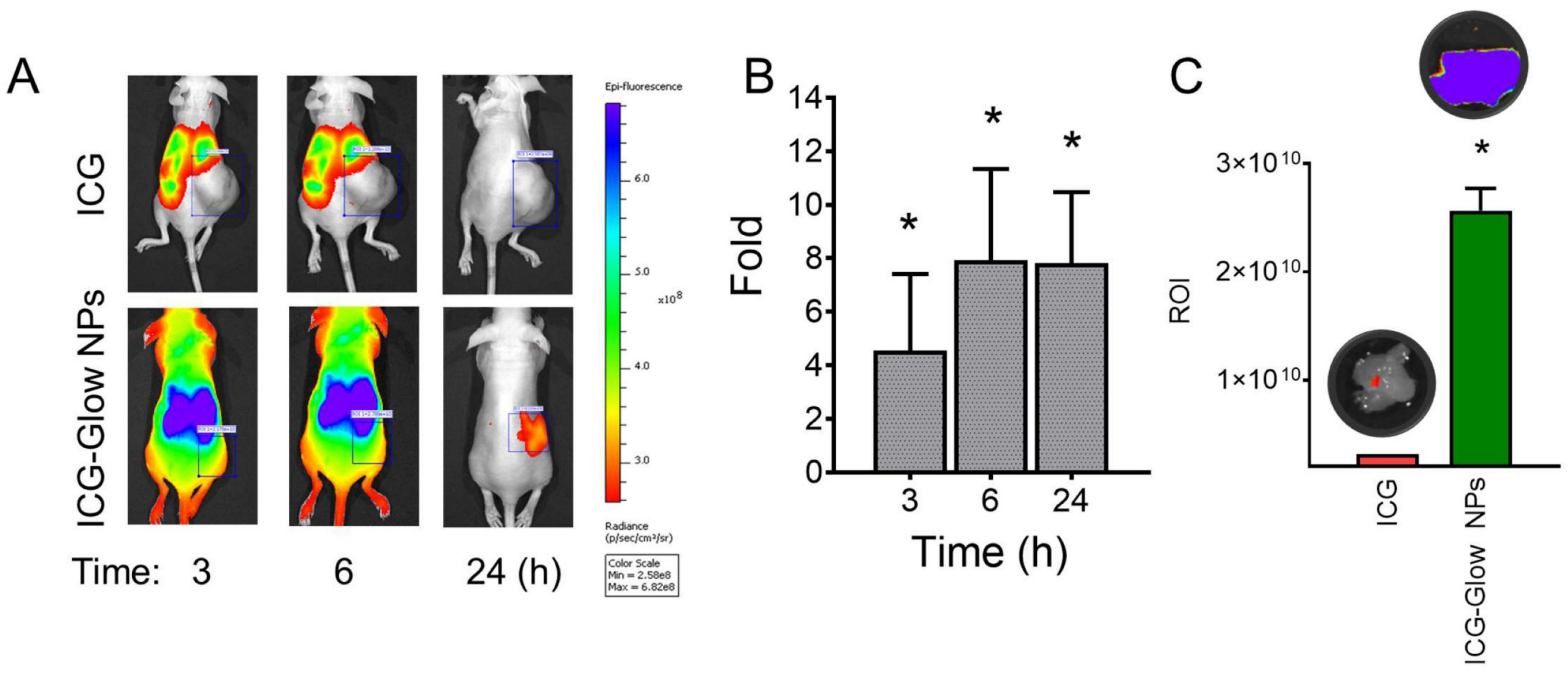
** Tumor-specific binding, targeting, and accumulation behavior of ICG-Glow NPs. A)** *In vivo* biodistribution and tumor specific delivery of ICG-Glow NPs in mice MDA-MB-231 xenograft tumors. The treatment is 100 μg of ICG or 100 μg of ICG equivalent ICG-Glow NPs. **B)** Superior tumor accumulation of ICG-Glow NPs in mice. **C)** Quantitative *ex vivo* analysis of the fluorescence signals of tumors (after euthanizing mice). Error bars show SEM, n=3. *p<0.05.

The enhanced tumor targeting behavior of ICG-Glow NPs was confirmed by live mice biodistribution and *ex vivo* tissue organ imaging analysis 51. The ICG group treated mice exhibited indistinguishable signals in tumors right from 3 h to 24 h, while ICG-Glow NPs evidently accumulate in tumors and are retained even at 24 h time point (**Figure 7A-B**). The fluorescence signals of *ex vivo* internal organs further verified the significant accumulation of ICG-Glow NPs in tumor tissues (**Figure 7C**). Altogether, this experiment verified that direct targeting of ICG-Glow NPs is due to the presence of tannic acid layered coating.

### Tannic acid in ICG-Glow NPs facilitates tumor cell specific binding and targeting

**Figure 8:**
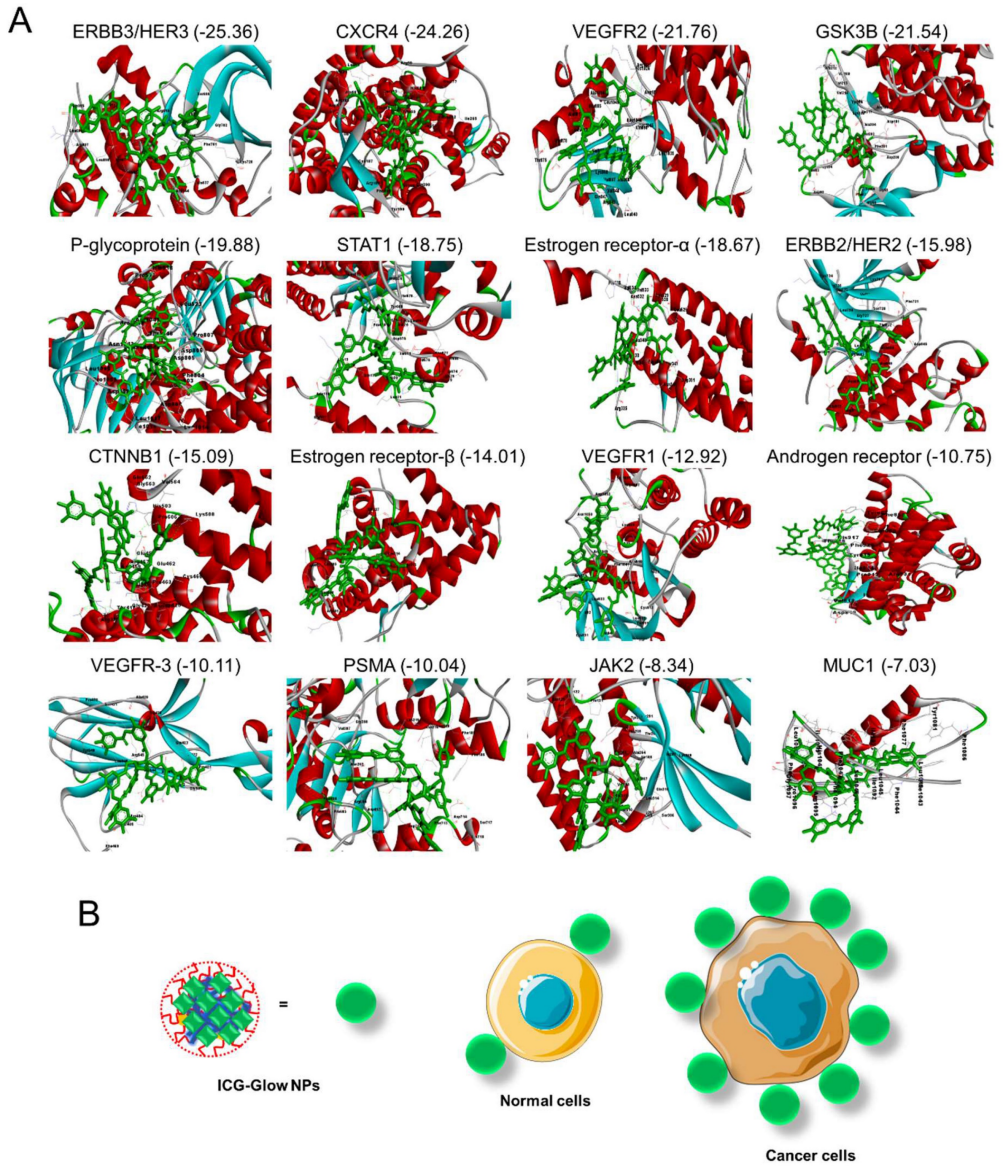
** Molecular interaction of tannic acid with oncogenic surface proteins.** Figures were produced utilizing Discovery Studio. **A)** Binding pattern of tannic acid (PubChem CID: 16129778) and various surface protein ERBB3/HER3, CXCR4, VEGFR2, GSK3B, P-glycoprotein, STAT1, ERBB2/HER2, CTNNB1, Estrogen Receptor-β, VEGFR1, Androgen Receptor, VEGFR-3, PSMA, JAK2, and MUC1. Docking was feasible using FireDock with -25.36 to -7.03 global binding energy, respectively. **B)** Tannic acid in ICG-Glow NPs facilitates tumor cell specific binding/targeting that enables tumor specific imaging characteristics.

Tannic acid is a widely studied molecule as a pharmaceutical excipient. A number of studies revealed that tannic acid inhibits oncogenic pathways by binding. Therefore, a docking analysis was performed on tannic acid and its interaction with prominent oncoproteins (cancer cell surface markers). **Figure 8** presents the interaction of tannic acid and 16 proteins with the global binding score range (-25.36 to -7.03).

All 16 proteins exhibited acceptable binding scores. The top ten binding proteins includes, ERBB3/HER3 (-25.36) showing the highest binding score with tannic acid followed by CXCR4 (-24.26), VEGFR2 (-21.76), GSK3B (-21.54), P-glycoprotein (-19.88), STAT1 (-18.75), estrogen receptor-α (-18.67), ERBB2/HER2 (-15.98), CTNNB1 (-15.09), and estrogen receptor-β (-14.01). All interaction patterns were mentioned in **Table S1 (Supplementary Information).**

## Discussion

Near-infrared fluorescence (NIRF) agents with higher sensitivity and photostability have gained prominent interest in the past years for cancer bioimaging applications in both preclinical and clinical settings 52. NIRF probes offer deep tissue penetration, low auto-fluorescence, easy portability and minimal light scattering, thus providing higher resolution 53. Thus far, ICG is the only clinically approved NIRF dye available for biomedical purposes 54 such as angiography, sentinel lymph node mapping, cardiac and hepatic function assessments, and intraoperative image guided surgery 55, 56. Despite having these many advantages, the clinical use of ICG for cancer applications is still restricted in cancer imaging. One of the biggest issues with ICG is that it is not cancer cell/tissue specific. Additionally, ICG is highly unstable due to its non-specific blood protein binding 19.

Tumor-specific delivery of NIR dyes or formulation improves the selection, identification, and accurate resection of tumor lesions during the surgical process 28. Currently, fluorescence-guided surgery is widely under development and a number of clinical trials have been open for specific detection of tumor lesions which otherwise are highly difficult to detect. Fluorescence-guided surgical approach prevents repeated surgeries and their associated morbidities. Such improved and accurate removal of tumors certainly improve the patient outcome and survival. Therefore, development of precise tumor targeted NIR fluorescent agents or formulation(s) can change the paradigm in fluorescence imaged guided surgery.

Herein, to improve the cancer specificity/targeting of an imaging agent, we have utilized PVP and TA-based nanoparticle system to generate fluorescently stable and cancer cell specific NIRF imaging probe. TA (naturally occurring compound) and PVP (chemically synthetic compound) are mainly used as pharmaceutical excipients that act as a stabilizer for hydrophobic agents and as an adhesive/binder for different formulation preparations, respectively. Following our established methodologies, we engineered self-assembled ICG-Glow NPs.

Various types of Nano-ICG formulations have been reported for tumor targeting or tumor visualization purposes 28. This study demonstrates that ICG when present in ICG-Glow NPs, is capable of improved imaging capabilities (**Figure 2, 5-7**). Initial DLS-based physico-chemical measurements confirmed that ICG-Glow NPs maintained an ideal small particle size and a firm negative zeta potential which are adequate for dense tumor tissue penetration and imaging (**Figure 1B-C**). More importantly, nanoparticle formation shifted ICG more toward the NIR region during the spectral analysis (**Figure 1D**). ICG-Glow NPs represented a constant concentration-dependent increase in fluorescence intensity (**Figure 2 A-B**). This behavior of ICG-Glow NPs was additionally observed in chicken breast tissue imaging (**Figure 2 C-D**), authenticating the improved photostability of ICG-Glow NPs in tissue(s).

The developed fluorescent imaging probe must be biocompatible and exhibit low cellular toxicity. Our ICG-Glow NPs indicated superior compatibility with red blood cells as the result of hemolysis evaluation, exhibiting minimal to no toxicity toward these cells (**Figure 3**). Similarly, ICG-Glow NPs did not show any toxicity or effect on the growth of epithelial normal cells (**Figure 4**). The reason for compatibility is due to non-toxic ingredients (PVP, TA, and ICG) in ICG-Glow NPs nanoformulation.

Recent advances in real-time fluorescence-based imaging and image-guided surgical options have led to the creation of novel agents or formulations that can efficiently distinguish cancerous cells/tissues from normal cells/tissues. Currently, the widely investigated formulations that often are composed of ligand-targeted components facilitate the binding to EGFR, PSMA, transferrin, folate receptors, etc., but most human cancers still cannot be imaged. Thus, we have explored a range of cancerous cell lines and TMAs to establish cancer specific imaging potential of ICG-Glow NPs. Following basic protocols (without antibody staining/probing) for immunofluorescence and immunohistochemistry, slides were prepared for *in vitro* (cancer cell lines) and *in situ* (tumor tissue microarrys) imaging. Our previously published work has already validated the cancer targeting/specificity of nanosystems 30, 32, in accordance with these findings, cells treated with ICG-Glow NPs were more fluorescently bright compared to free ICG (**Figure 6**). Supporting this information further, ICG-Glow NPs displayed higher binding affinity to cancerous tissues than that of free ICG (**Figure 5**). The fluorescence signal emitted by ICG-Glow NPs probed tumor tissues were more intense than those stained with free ICG in all incidences. The prime reason for enhanced tumor specificity of ICG-Glow NPs is due to the presence of tannic acid in the formulation. Multiple oncogenic signaling cascades (EGFR, CXCR4, FASN, P-gp, β-catenin, GSK-3B, STAT1, JAK2, MUC1, etc) are hallmarks of cancer cells that are associated with drug resistance, tumorigenic potential, and metastasis. Tannic acid is a well-known EGFR 57 and FASN (even at 0.15 µM concentration) 58 inhibitor. Tannic acid when decorated onto magnetic nanoparticles enabled their tumor specific binding due to the polyphenol structure of TA and the glycocalyx on cancer cells 59. Such TA modified construct was able to capture up to 93% of tumor cells while sparing normal cells. Therefore, we hypothesized that TA on the ICG-Glow NPs efficiently binds to EGFR, CXCR4, FASN, and other oncoproteins (**Figure 8**), thus facilitating tumor specific binding/targeting (**Figure 5-7**) which can further provide a novel option for tumor specific probe development.

As previously mentioned, there are several ICG-based contrast agents under clinical trials for tumor detection 60. Among them, pegsitacianine, ICG-Chlorin-e6 nanocluster, and poly(caprolactum)-ICG micelles are well studied. However, still, the targeted delivery of the fluorescent dyes to the tumors is challenging. Thus, a new strategy is urgently needed to promote translational cancer nanoimaging. Only certain cancers can be targeted by monoclonal antibody conjugated agents 61, 62. However, ICG labelling is required at higher concentrations to conjugate onto antibodies otherwise, signals will be weak. Extensive conjugation may result in poor targeting of cancer cell. Thus, ICG-Glow NPs approach is capable of encapsulating larger amounts of ICG, making it suitable for image guided applications. Altogether, this proof-of-concept data signifies that our novel ICG-Glow nanoformulation could be utilized as a prime contrast bioimaging agent, promising a stable NIRF bioimaging platform for deep tissue visualization. However, further *in vivo* investigations are warranted to understand the bioavailability, biodistribution, tumor specific targeting, and clearance profiles of ICG-Glow NPs system.

## Conclusion

Information obtained from this study strongly suggests the potential of ICG-Glow NPs becoming a lead contrast agent for NIRF-based cancer bioimaging. ICG-Glow NPs indicated ideal particle size and surface charge for cancer cell/tissue delivery. ICG-Glow NPs presented a dose-dependent fluorescence intensity, even for *ex vivo* tissue imaging. In addition, ICG-Glow NPs are highly hemo- and cellular-compatible and displayed a selective cancer cell/tissue binding/affinity compared to free ICG. Herein, this piece of work promises the possibility of ICG-Glow NPs for becoming a biocompatible, safe-to-use, cancer specific NIR fluorescence bioimaging platform for deep tissues in pre-clinical and clinical settings.

## Supplementary Material

Supplementary figures and table.
