# Supplementary material for: Indocyanine Green-based Glow Nanoparticles Probe for Cancer Imaging
Source: Nanotheranostics. 2023 Apr 9;7(4):353–67. doi: 10.7150/ntno.78405 (PMC10161388; doi:10.7150/ntno.78405)
Supplement: Supplementary file 1 — Supplementary figures and table. [file ntnov07p0353s1.pdf]

Supplementary Information

Indocyanine Green-based Glow Nanoparticles Probe for Cancer Imaging

Neeraj Chauhan[1,2], Marco Cabrera[1,2], Pallabita Chowdhury[3], Prashanth K.B. Nagesh[1,3,4], Anupam Dhasmana[1,2], Pranav[1,2], Meena Jaggi[1,2], Subhash C. Chauhan[1,2], Murali M. Yallapu[1,2*]

[1]Department of Immunology and Microbiology, School of Medicine, The University of Texas Rio Grande Valley, McAllen, TX 78504, United States

[2]South Texas Center of Excellence in Cancer Research, School of Medicine, The University of Texas Rio Grande Valley, McAllen, TX 78504, United States

[3]Department of Pharmaceutical Sciences, University of Tennessee Health Science Center, Memphis, Tennessee 38163, United States

[4]Laboratory of Signal Transduction, Memorial Sloan Kettering Cancer Center, New York, New York 10065, United States

*Corresponding author:

Murali M. Yallapu, Ph.D.
Associate Professor
Department of Immunology and Microbiology
School of Medicine
University of Texas Rio Grande Valley
McAllen, TX, 78504, USA
Tel.: +1 (956) 296 1734
E-mail: murali.yallapu@utrgv.edu

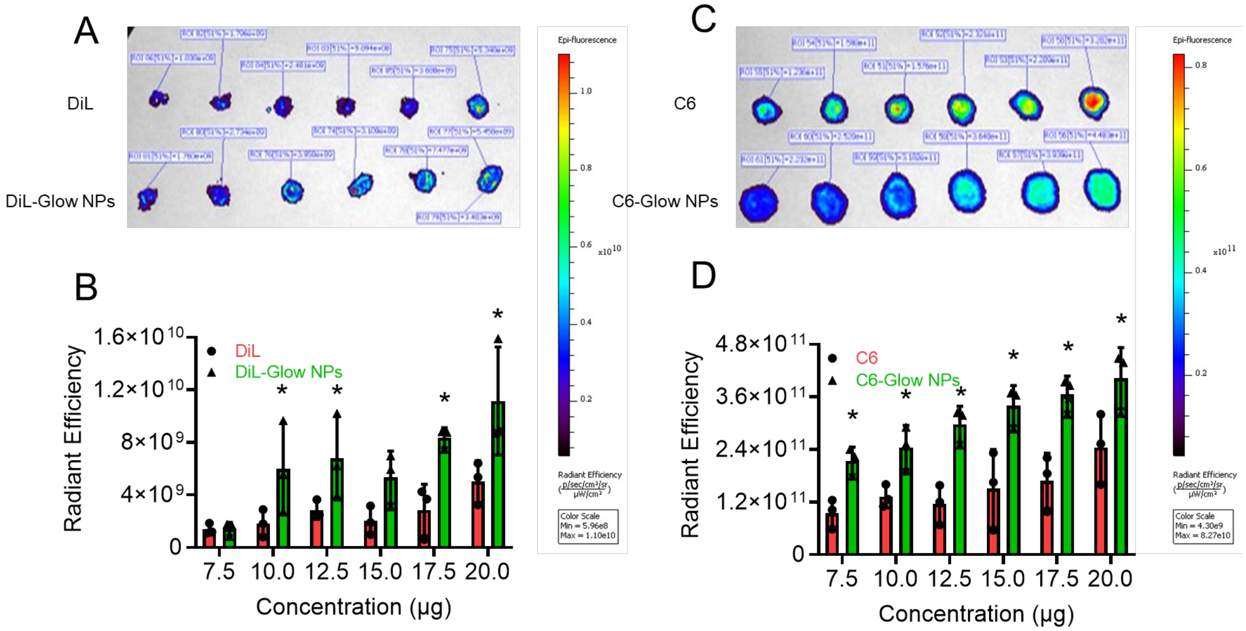

Figure S1. NIR fluorescence imaging characteristics of DiL-Glow NPs and C6-Glow NPs. A,C) DiL, DiL-Glow NPs, C6, and C6-Glow NPs fluorescence measurement shown on filter paper image, and **B,D)** Quantitative analysis of the fluorescence signals ROI (radiation efficiency = photons/sec/cm^2/sr) of samples. Data represents the average of three individual experiments. Error bars show SEM, n=3. *$p<0.05$.

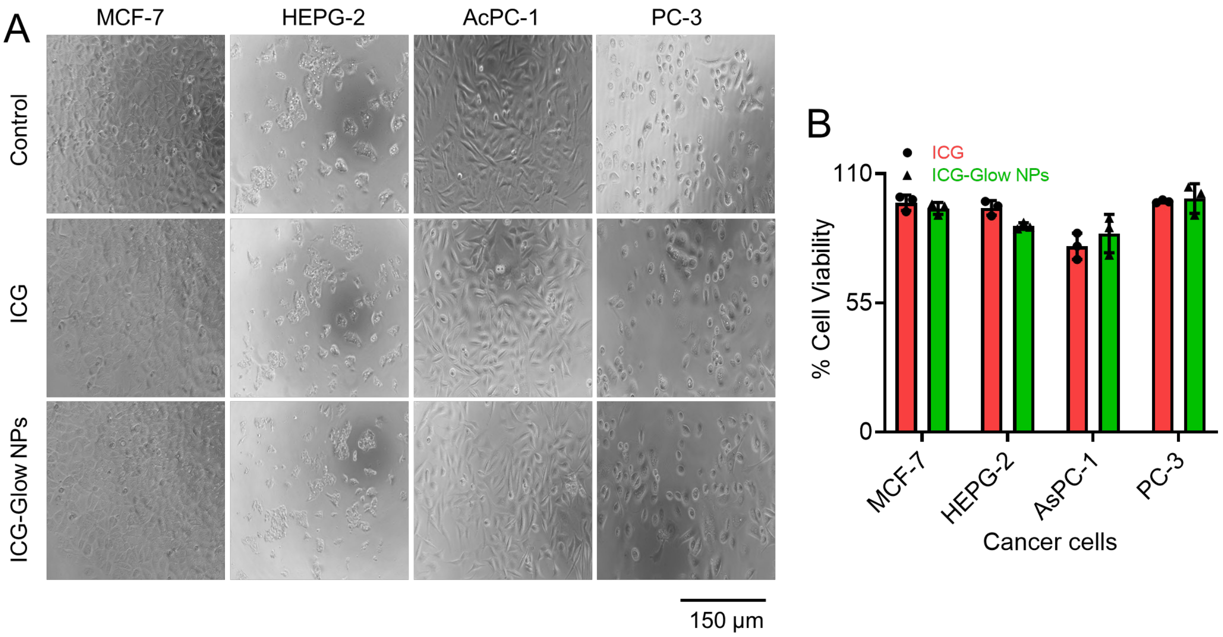

Figure S2. Cellular-biocompatibility of ICG-Glow NPs. A-B) Cellular biocompatibility of ICG-Glow NPs on various cancer cell lines using an MTS cell proliferation assay. MCF-7 (Breast cancer), HEPG-2 (Liver cancer), AsPC-1 (Pancreatic cancer), and PC-3 (Prostate cancer) cell lines were treated with 100 µg free ICG and ICG-Glow NPs for 24 h and absorbance was measured after MTS reagent supplementation at 490 nm to determine cell viability. Results were normalized to non-treated control cells. Error bars show SEM, n=3. *$p<0.05$.

Table 1: Binding interaction affinity and pattern analysis of various biomolecules and tannic acid.

Protein	PatchDock Score	FireDock Score	Binding Residues	H-Bond
ERBB3/HER3 PDB: 3LMG	9096	-25.36	Ser698, Gly699, Val700, Phe701, Gly702, Lys728, Arg814, Arg819, Ala837, Asp844, Thr854, Pro855, Ile856, Lys857, Met859, Tyr869, Glu891, Gly895, Leu896, Arg897, Leu898	SER698:OG - TA:O73; VAL700:N - TA:O62 LYS728:NZ - TA:O51; ARG814:NH2 - TA:O96; LYS857:NZ - TA:O117; LEU896:N - TA:O7; TA:O7 - LEU896:O; TA:O73 - SER698:OG; TA:O73 - GLY699:O; TA:O118 - GLU891:OE1
CXCR4 PDB: 4RWS	10440	-24.26	Arg30, Asn37, Leu41, Asp97, Ala98, Val99, Tyr121, Arg183, Ile185, Cys187, Arg188, Phe189, Tyr190, Phe199, Gln200, His203, Tyr255, Ile259, Asp262, Ile265, Glu277, His281, Ile284, Ser285, Glu288	ARG30:NE - TA:O39; ARG30:NE - TA:O51 ARG30:NH1 -TA:O39; ARG30:NH2 - TA:O39; ARG30:NH2 - TA:O71; ARG183:NH2 -TA:O94; CYS187:SG - TA:O75; ARG188:NE - TA:O118; ARG188:NH1 - TA:O118; ARG188:NH2 - TA:O118; TYR190:OH - TA:O118; TYR190:OH - TA:O119; GLN200:N - TA:O8 HIS203:ND1 - TA:O6; HIS203:ND1 - TA:O7 TYR255:OH - TA:O21; HIS281:ND1 - TA:O29; HIS281:ND1 - TA:O30; HIS281:ND1 - TA:O32; TA:O18 - TA:O10 TA:O71 - TA:O39; TA:O78 - TA:O55 TA:O96 - ALA98:O; TA:O98 - SER285:O TA:O98 - GLU288:OE2; TA:O118 - TYR190:OH ; TA:O119 - TYR190:OH
VEGFR2 PDB: 2P2H	9040	-21.76	Leu840, Arg842, Gln847, Val848, Ala866, Val867, Lys868, Thr875, Glu878, Ala881, Leu882, Glu885, Val899, Val914, Thr916, His1026, Arg1027, Asp1028, Leu1035, Cys1045, Asp1046, Leu1049, Ala1050	LYS868:NZ - TA:O110; THR916:OG1 - TA:O118; ARG1027:NH2 - TA:O96; ASP1028:N - TA:O98; CYS1045:SG - TA:O117; TA:O72 - GLU878:OE2; TA:O98 - ARG1027:O; TA:O117 - CYS1045:SG TA:O118 - THR916:OG1
GSK3B PDB: 1PYX	9148	-21.54	Gly65, Ser66, Phe67, Gly68, Asp90, Phe93, Lys94, Arg96, Glu97, Arg180, Asp181, Asp200, Phe201, Gly202, Ser203, Ala204, Lys205, Gln206, Val208, Glu211, Pro212, Asn213, Val214, Tyr216, Ile217	ARG96:NH1 - TA:O99; ARG96:NH2 - TA:O32; ARG96:NH2 - TA:O41; ARG180:NH1 - TA:O121; ARG180:NH2 - TA:O120; ARG180:NH2 - TA:O121; TA:O19 - ALA204:O; TA:O50 - GLU211:OE2; TA:O71 - GLU211:OE1; TA:O72 - GLU211:OE1; TA:O72 - PRO212:O TA:O97 - ASP90:OD1
P-glycoprotein PDB: 6C0V	9922	-19.88	Pro517, His518, Leu523, Asp800, Ser802, Trp803, Phe804, Asp805, Asp806, Pro807, Lys1014, Leu1017, Ile1018, Asn1043, Tyr1044, Pro1045, Thr1046, Arg1047, Pro1048, Asp1088, Pro1089, Leu1090	LEU523:N - TA:O118; SER802:OG - TA:O94; TRP803:NE1 - TA:O73; ASP805:N - TA:O75; ASP806:N - TA:O74 ASP806:N - TA:O75; LYS1014:NZ - TA:O72 LYS1014:NZ - TA:O73; TA:O96 - ASP1088:OD1; TA:O96 - ASP1088:OD2 TA:O120 - ASP806:OD1; TA:O120 - ASP806:OD2; TA:O121 - ASP806:OD2
STAT1 PDB: 1YVL	9366	-18.75	Lys13, Gln17, Gln20, Leu21, Glu117, Asn118, Arg512, Asn515, Gln518, Asn574, Asp575, Gly576, Cys577, Ile578, Met579, Leu667, Tyr668, Pro669, Ile671,	GLN17:NE2 - TA:O62; ARG512:NE - TA:O94; ARG512:NH2 - TA:O94; CYS577:N - TA:O78; CYS577:SG - TA:O78 LYS679:NZ - TA:O108; LYS679:NZ - TA:O110; TYR680:N - TA:O117; TA:O72 -

			Asp674, His675, Ala676, Phe677, Gly678, Lys679, Tyr680	ASN118:OD1; TA:O75 - ASP575:OD1; TA:O117 - LYS679:N
Estrogen receptor-a PDB: 1A52	7286	-18.67	Arg335, Ser338, Ala340, Ser341, Gly344, Thr347, Asn348, Asp351, Leu525, Met528, Lys529, Asn532, Val533, Val534, Pro535	ARG335:NE - TA:O98; ARG335:NH1 - TA:O21; SER338:OG - TA:O97; LYS529:NZ - TA:O50; TA:O48 -ASP351:OD2; TA:O49 - ASP351:OD1; TA:O49 - ASP351:OD2; TA:O51 - MET528:O; TA:O51 - ASN532:OD1; TA:O74 - AL533:O; TA:O97 - SER338:OG
ERBB2/HER2 PDB: 3RCD	9274	-15.98	Lys724, Val725, Leu726, Gly727, Ser728, Phe731, Val734, Lys736, Lys753, Gly804, Cys805, Leu807, Asp808, Arg811, Glu812, Asp845, Arg849, Asn850, Leu852, Thr862, Asp863, Phe1004, Ser1007	VAL725:N - TA:O117; LYS736:NZ - TA:O6; ARG811:NH1 - TA:O39; ARG811:NH1 - TA:O50; ARG811:NH1 - TA:O71; ASN850:ND2 - TA:O96; TA:O48 - GLU812:OE1; TA:O96 - ASP845:OD2; TA:O96 - ASP863:OD2; TA:O117 - VAL725:O
CTNNB1 PDB: 1JPW	7942	-15.09	Arg386, Ile414, Asn415, Thr418, Gly422, Ser425, Asn426, Arg457, Glu458, Asp459, Ile460, Glu462, Pro463, Cys466, His503, Pro505, Lys508, Glu562, Gly563, Val564	ASN415:N - TA:O6; ASN415:N - TA:O7 THR418:OG1 - TA:O6; ASN426:ND2 - TA:O98; ARG457:NE - TA:O118; LYS508:NZ - TA:O95; LYS508:NZ - TA:O96; TA:O6 - ASN415:OD1; TA:O6 - THR418:OG1; TA:O7 - ASN415:OD1; TA:O75 - GLU458:OE1; TA:O75 - GLU458:OE2; TA:O78 - GLU462:OE2; TA:O94 - PRO505:O; TA:O97 - ASN426:OD1
Estrogen Receptor Beta PDB: 5TOA	8028	-14.01	Ile310, Ser311, Lys314, Lys315, Val320, Leu324, Gln327, Val328, Leu495, Asn496, Ala497, His498, Val499, Leu500, Arg501	LYS314:NZ - TA:O32; ARG501:N - TA:O108; TA:O7 - ALA497:O; TA:O48 - SER311:O; TA:O52 - VAL320:O; TA:O97 - LEU324:O; TA:O120 - ARG501:O
VEGFR1 PDB: 3HNG	8328	-12.92	Lys831, Ser832, Leu833, Arg835, Gln843, Tyr911, Cys912, Lys913, Gly915, Asn916, Ser918, Asn919, Lys922, Leu1029, Phe1041, Arg1045, Asn1050, Asp1052, Glu1091, Gly1096, Gly1097, Ser1098	LYS831:NZ - TA:O94; LYS831:NZ - TA:O95; SER918:OG - TA:O6; ASN919:ND2 - TA:O110; ARG1045:NH1 - TA:O21; SER1098:N - TA:O7; SER1098:OG:B - TA:O7; TA:O6 - SER918:OG; TA:O6 - GLU1091:OE2; TA:O7 - SER1098:OG:B; TA:O18 - ASN1050:OD1; TA:O52 - SER832:O; TA:O95 - GLN843:OE1; TA:O97 -CYS912:O
Androgen Receptor PDB: 2PIW	7498	-10.75	Ile816, Pro817, Val818, Asp819, Gln824, Asp828, Arg831, Phe856, Tyr857, Thr860, Lys861, Pro913, Ile914, Tyr915, Phe916, His917, Thr918	ARG831:NH2 - TA:O120; HIS917:ND1 - TA:O71; THR918:N - TA:O41; TA:O119 - ASP828:OD1; TA:O121 - PHE916:O; TA:O18 - PRO913:O; TA:O7 - VAL818:O TA:O7 - PRO817:O
VEGFR-3 PDB: 4BSJ	7854	-10.11	Pro421, Pro422, Gln423, Lys427, Glu428, Ala429, Ser431, Pro432, Phe469, Pro484, Gln485, Gly541, Gln542, Asp543, Glu544, Arg545, Leu546, Ile547, Tyr548	GLN423:NE2 - TA:O6; LYS427:NZ - TA:O55; AGLN542:N - TA:O118; GLN542:N - TA:O119; TA:O52 - TYR548:OH; TA:O73 - GLN485:O; TA:O74 - GLU544:OE1; TA:O75 - GLU544:O; TA:O75 - GLU544:OE1; TA:O94 - LEU546:O; TA:O117 - PRO422:O; TA:O118 - GLN542:O; TA:O119 - GLN542:O
PSMA PDB: 7BFZ	9266	-10.04	Thr182, Phe185, Phe186, Lys215, Asn216, Leu219, Asn262, Arg281, Val287, Gly288, Pro682, Asp683, Arg684, Pro685, Phe686, Ser696, His697, Asp710,	LYS215:NZ - TA:O19; ASN262:ND2 - TA:O108; LYS718:NZ - TA:O94; ALA724:N - TA:O95; TA:O50 - HR182:O; TA:O51 - ASP714:OD2; TA:O73 - HIS697:NE2; TA:O74 - ASP710:OD1;

			Phe713, Asp714, Ser717, Lys718, Asp720, Lys723, Ala724	TA:O74 - ASP710:OD2; TA:O119 - ASN262:O
JAK2 PDB: 6E2Q	8940	-8.34	Pro121, Arg122, Asn129, Ile166, Lys167, Val168, Pro169, Gln262, Ser263, Ala264, Phe265, Tyr266, Thr267, Lys269, Thr291, Gln297, Ser306, Glu307, Leu309, Thr310, Glu311, Gln312, Leu314, Gln315	SER263:OG - TA:O11; ALA264:N - TA:O6; LYS269:NZ - TA:O10; TA:O48 - GLU311:O; TA:O48 - LEU314:O
MUC1 PDB: 6BSC	9704	-7.03	Phe1043, Phe1044, Leu1045, Ser1046, Phe1047, His1048, Ile1049, Leu1052, Phe1054, Ile1073, Phe1077, Tyr1081, Phe1086, Leu1089, Ile1092, Lys1093, Phe1094, Arg1095 Pro1096, Gly1097	LYS1093:H - TA:O22; ARG1095:HE - TA:O30; TA:O49 - ILE1049:O; TA:O49 - LEU1052:O; TA:O50 - LEU1052:O; TA:O51 - PRO1096:O; TA:O75 - SER1046:OG:B; TA:O117 - PHE1043:O
